# Supplementary material for: Data-driven identification of predictive risk biomarkers for subgroups of osteoarthritis using interpretable machine learning
Source: Nat Commun. 2024 Apr 1;15:2817. doi: 10.1038/s41467-024-46663-4 (PMC10985086; doi:10.1038/s41467-024-46663-4)
Supplement: Supplementary file 1 — Supplementary Information [file 41467_2024_46663_MOESM1_ESM.pdf]

Supplementary information for

Data-driven identification of predictive risk biomarkers for subgroups of osteoarthritis using interpretable machine learning

Supplementary Figures

Supplementary Fig. 1

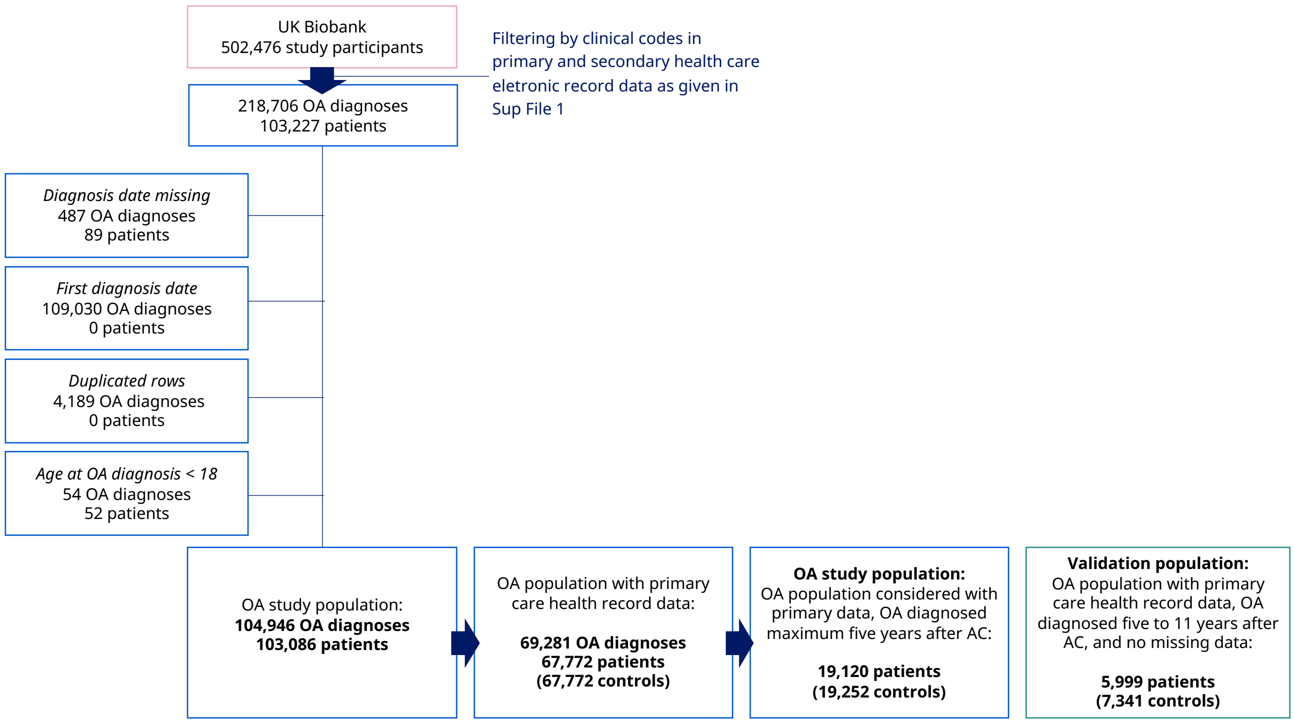

Supplementary Fig. 1: OA study population in UK Biobank. Clinical codes of OA were mined in primary care (read2/read3 (CTV3)) and secondary care (ICD-9/ICD-10) data. Any trauma-induced and nonsense OA diagnoses codes were excluded for identification of OA diagnoses and patients. Following inclusion criteria 103,086 patients diagnosed with OA were identified (corresponding to 20.5% of the entire UK Biobank population). Individuals without a clinical diagnosis of OA (N=399,249) were controlled for follow-up in the death register. If a death date were registered, the individual was excluded from analysis to ensure follow-up during the entire study period. Furthermore, they were required to have available linked primary health care data to enable capture of longitudinal features in the machine learning models. This left N=153,028 individuals eligible for selection as a control. Based on the number of available cases, a similar number of controls were randomly selected and matched 1:1 with OA cases to get a matched index date (N=67,772). Further filtering included that the OA diagnosis date(cases)/matched index date(controls) was maximum five years after the UK Biobank recruitment assessment centre (2006-2010).

Supplementary Fig. 2

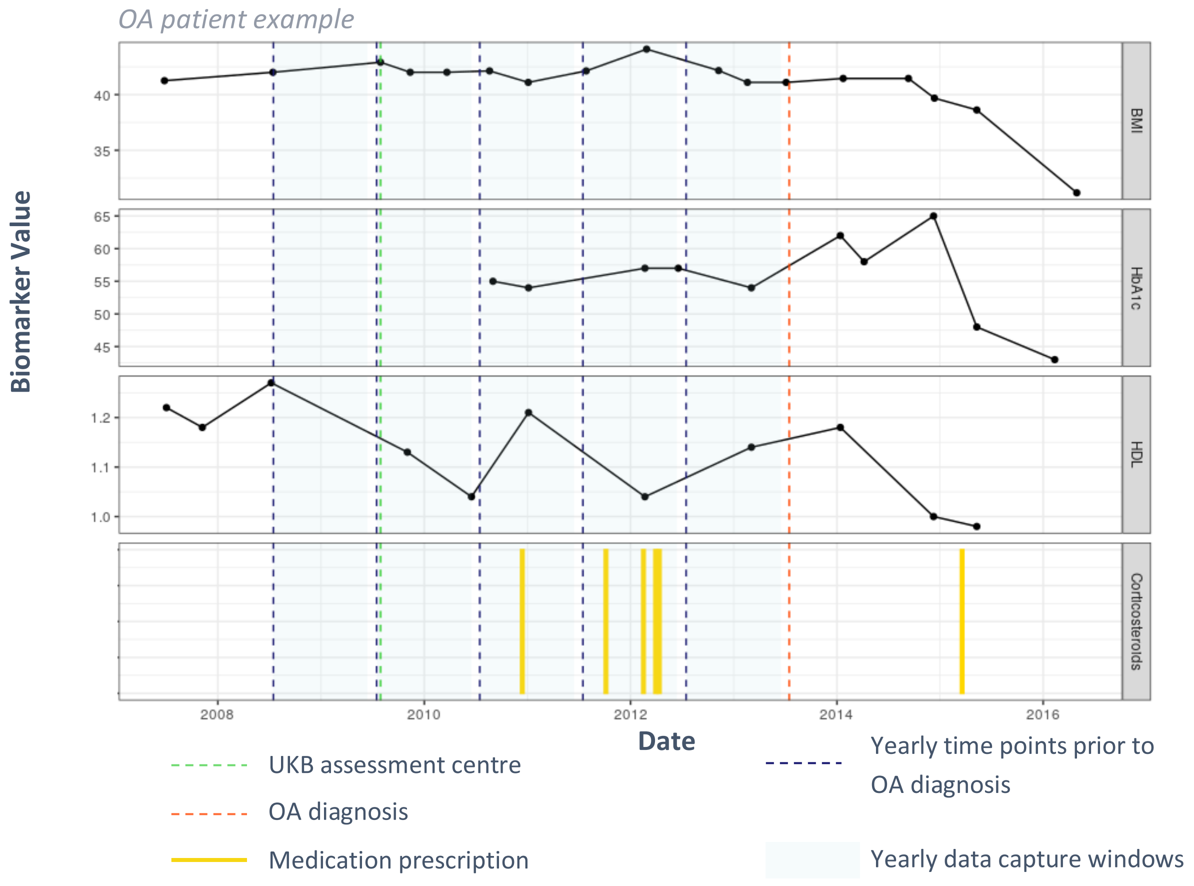

Supplementary Fig. 2: Yearly binning of longitudinal patient data. Longitudinal patient data was captured from primary care GP data and is shown for an example patient with OA. Date of UK Biobank (UKB) assessment centre is shown with the green dotted line and is required to be no more than 5 years prior to the date of OA diagnosis, shown by the red dotted line. In the 5 years prior to OA diagnosis, 5 yearly data capture windows are established (shown by blue dotted lines and blue shaded regions). For clinical biomarkers, the average value in each yearly data capture window is extracted. BMI given as Kg/m2, HbA1c given as mmol/mol, and HDL given as mmol/L. For drug prescriptions, a binary value (0/1) is assigned for each data capture window, reflecting whether a drug had been prescribed in this yearly time-period (indicated by a yellow bar). Longitudinal data extractions were then used as input features to the XGBoost models.

Supplementary Fig. 3

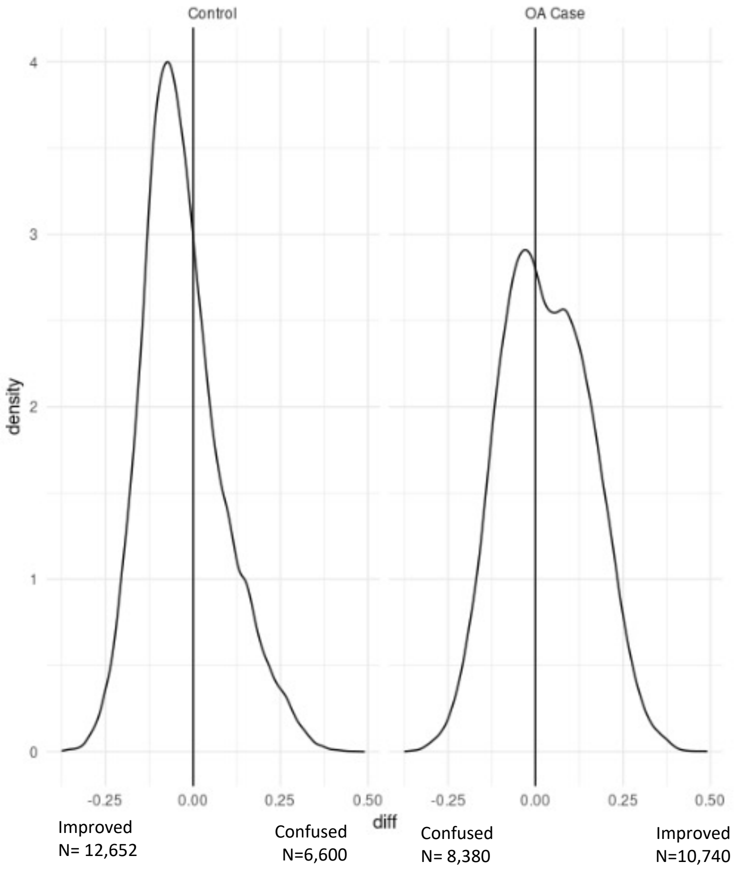

Supplementary Fig. 3: Density plot of difference in individual predicted risk scores between OA risk predicted by the Clin model subtracted by the individual risk scores of OA risk model only including age, sex and BMI (x-axis: diff = difference between prediction probability score generated by the model stratified by observed Control (left plot) or Case (right plot) status)). Vertical black lines at 0 represent an agreement between the Clin model and the simpler model based on age, sex and BMI in predicted risk score. A two-sided t.test comparing distribution between the predicted probabilities showed significantly different predicted distributions by a simple model (age, sex, BMI) vs Clin model both for cases and controls (both had p-value <2e-16).

Supplementary Fig. 4

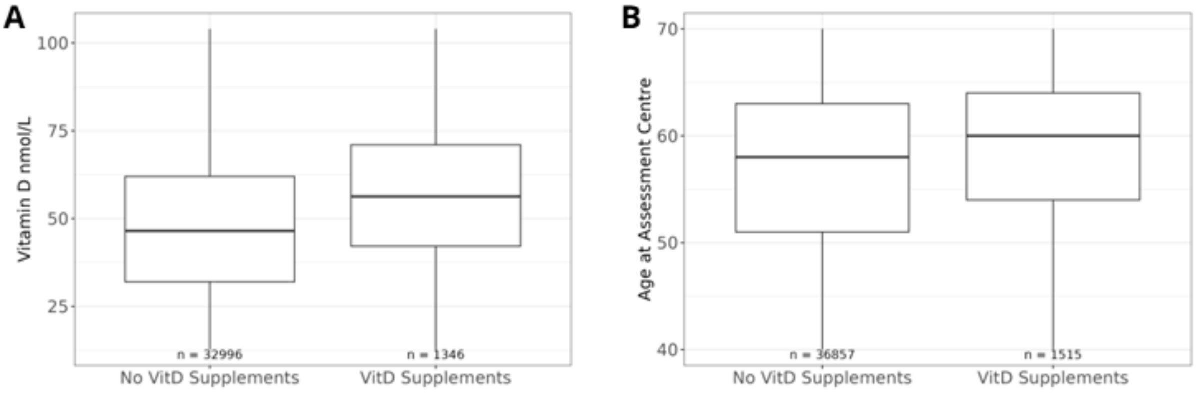

C

**Characteristic**	**No OA**, N = 15,874[1]	**OA**, N = 15,203[1]
**Had_menopause_0**		
Had menopause	5.54% (274/4,943) [4.9-6.2]	6.18% (409/6,613) [5.6-6.8]
Have not had menopause	2.74% (79/2,880) [2.2-3.4]	3.45% (49/1,422) [2.6-4.6]
Male	2.53% (204/8,051) [2.2-2.9]	2.71% (194/7,168) [2.3-3.1]
**Overall**	3.51% (557/15,874) [3.2-3.8]	4.29% (652/15,203) [4.0-4.6]

[1] Proportion (%) of participants using vitamin D supplements (n/N) [95% CI]

Supplementary Fig. 4: Vitamin D supplements. Cases of OA and controls segregated by those who report taking vitamin D supplements and those who do not report taking vitamin D supplements at assessment centre. 4A) Their associated vitamin D levels at assessment centre (p-value < 2.2e-16 in two-sided t.test). 4B) Their age at assessment centre (p-value < 2.2e-16 in two-sided t.test). 4C) Table of study participants that take vitamin D supplements stratified by menopause status. The confidence intervals were calculated using the Clopper-Pearson exact method.

Supplementary Fig. 5

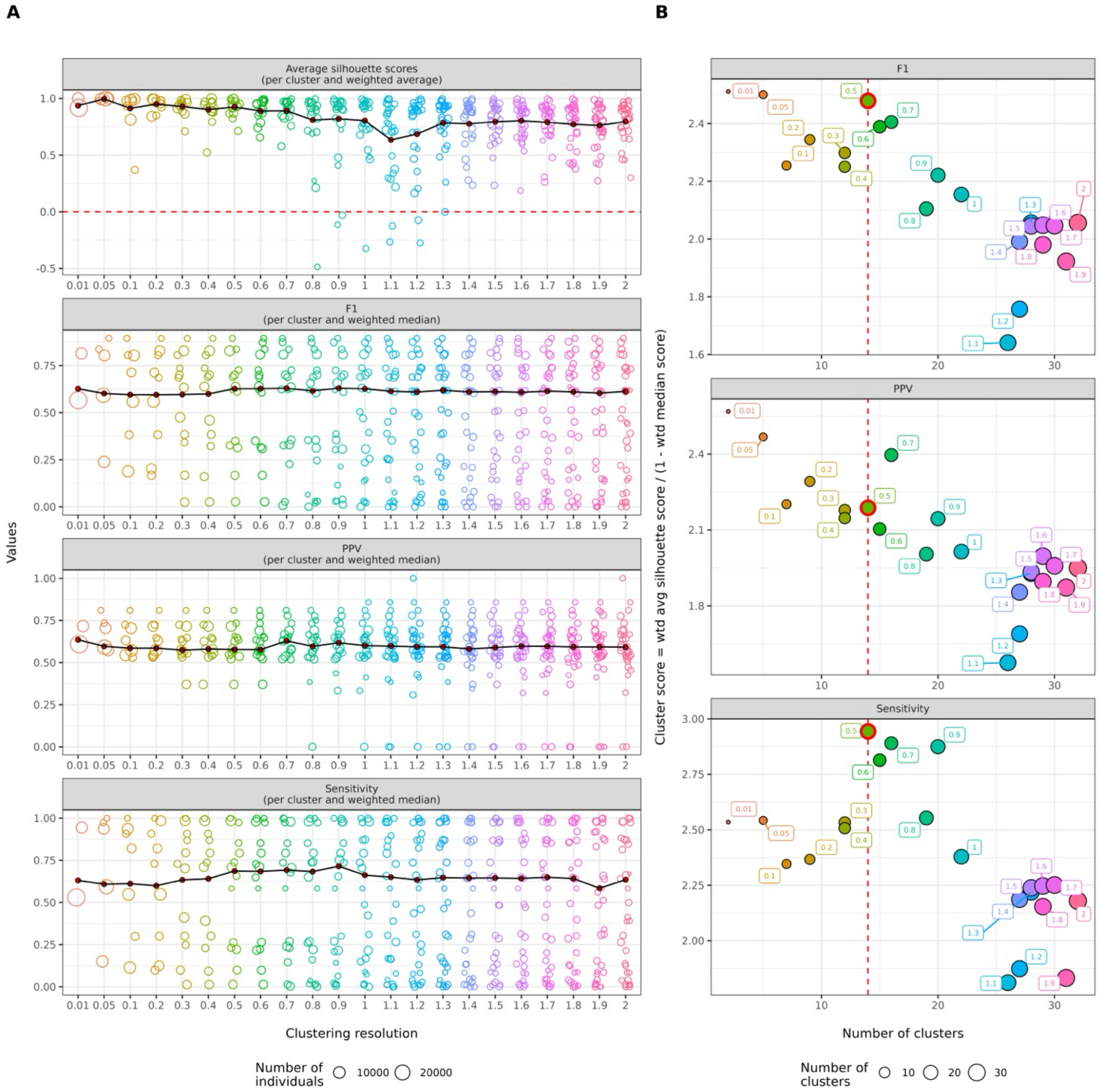

Supplementary Fig. 5: Clustering resolution optimisation. 5A) Silhouette scores (cluster robustness) as well as F1/Precision (PPV)/Recall were determined for each cluster at various clustering resolution, and the weighted mean and medians (respectively) calculated per resolution. 5B) A cluster score was defined to integrate the silhouette scores and prediction metrics per resolution to determine which resolution leads to an optimisation of the number of high-quality clusters and clusters with good predictive value. Colours and labels indicate the clustering resolution. The red dotted line indicates the chosen cluster resolution (resolution = 0.5, 14 clusters).

Supplementary Fig. 6

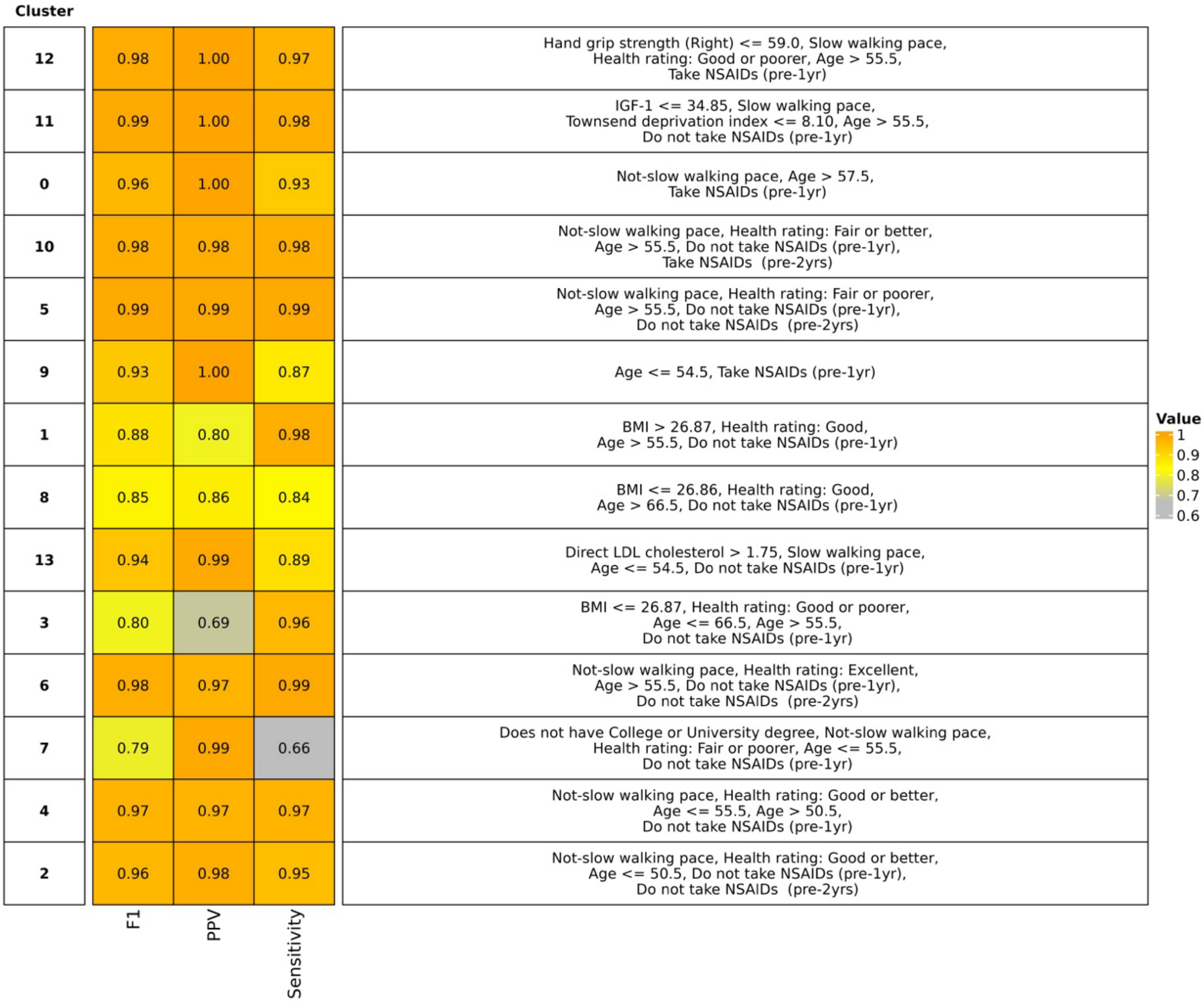

Supplementary Fig. 6: Cluster rules performance. Out-of-bag performance values (F1/precision/recall) for each cluster. These values indicate how accurately each set of rules attribute individuals to each cluster. Cluster sample sizes provided in Supplementary Data 3.

Supplementary Fig. 7

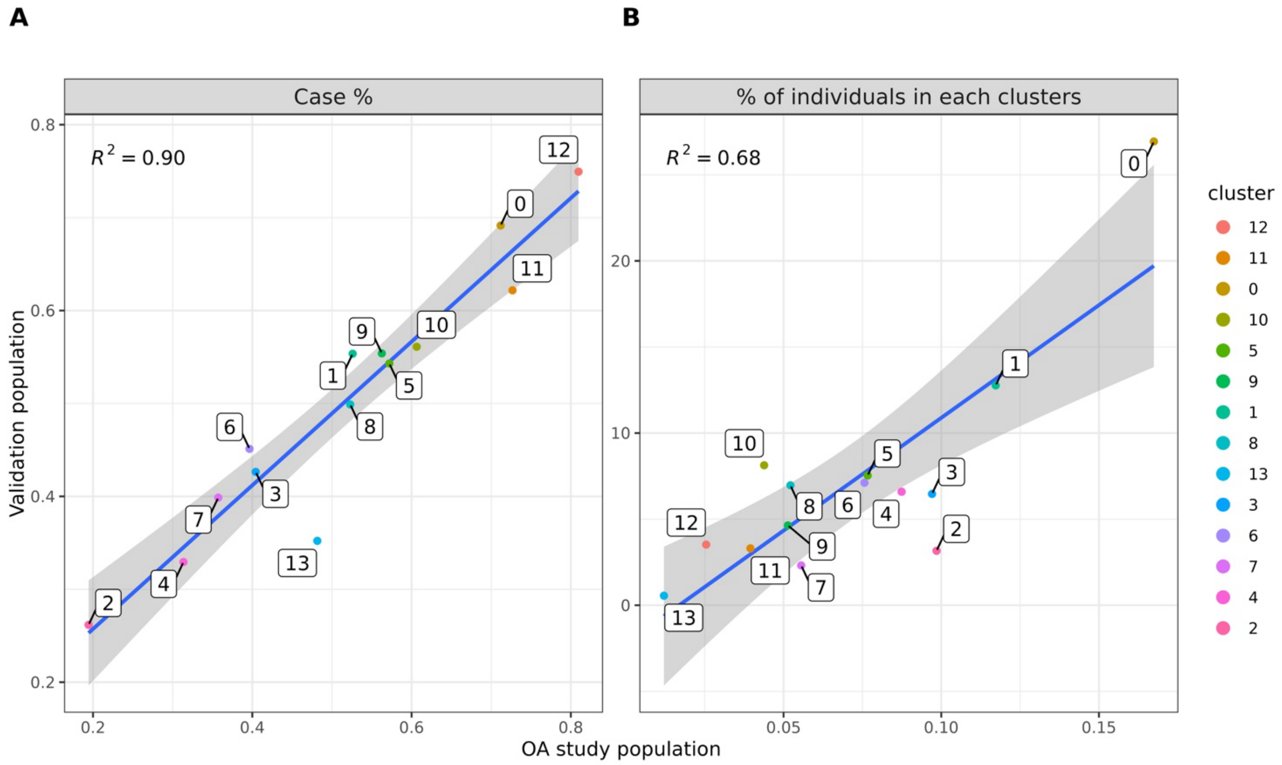

Supplementary Fig. 7: Correlations between OA study and validation populations. 7A) Percentage of cases in each clusters. 7B) Percentage of individuals in each cluster. Individuals in the OA study population were clustered based on SHAP value (cf. Fig. 4); individuals in the validation population were attributed to each cluster following the cluster-defining rules described in Fig. 5.

Supplementary Fig. 8

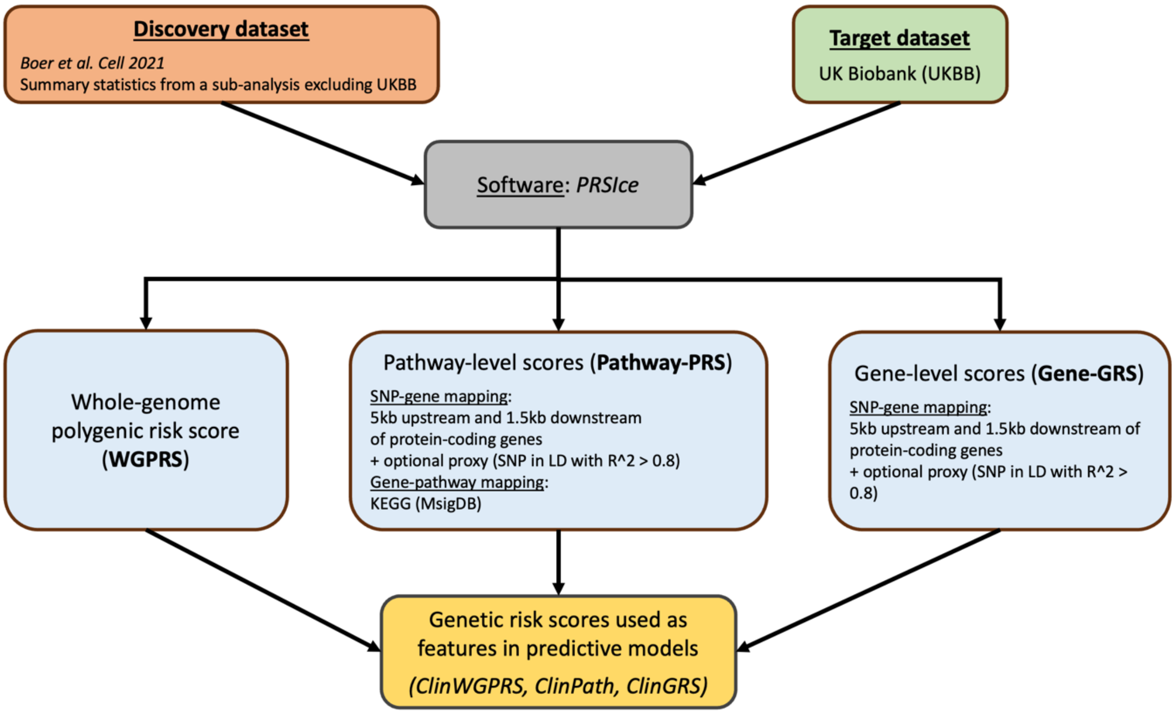

Supplementary Fig. 8: Schematic representation of the methods for the generation of genetic risk scores. Single-nucleotide polymorphisms (SNPs) were aggregated at the whole-genome-level, biological pathway-level (KEGG pathways) and gene-level to use as genetic features in the osteoarthritis predictive model.

Supplementary Fig. 9

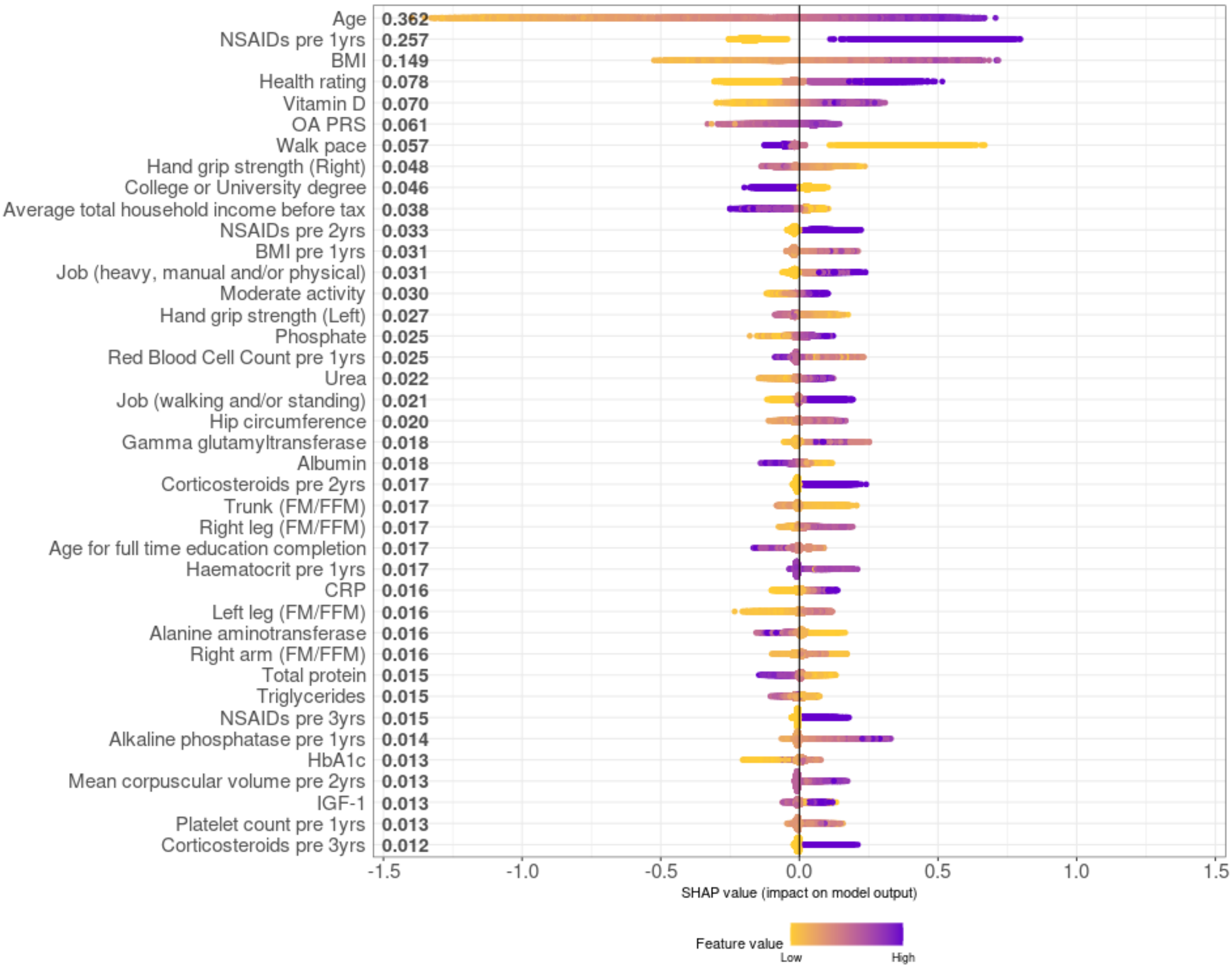

Supplementary Fig. 9: Ranked feature importance of ClinWGPRS model by SHAP additive explanations for top 40 predictive features in the model.

Supplementary Fig. 10

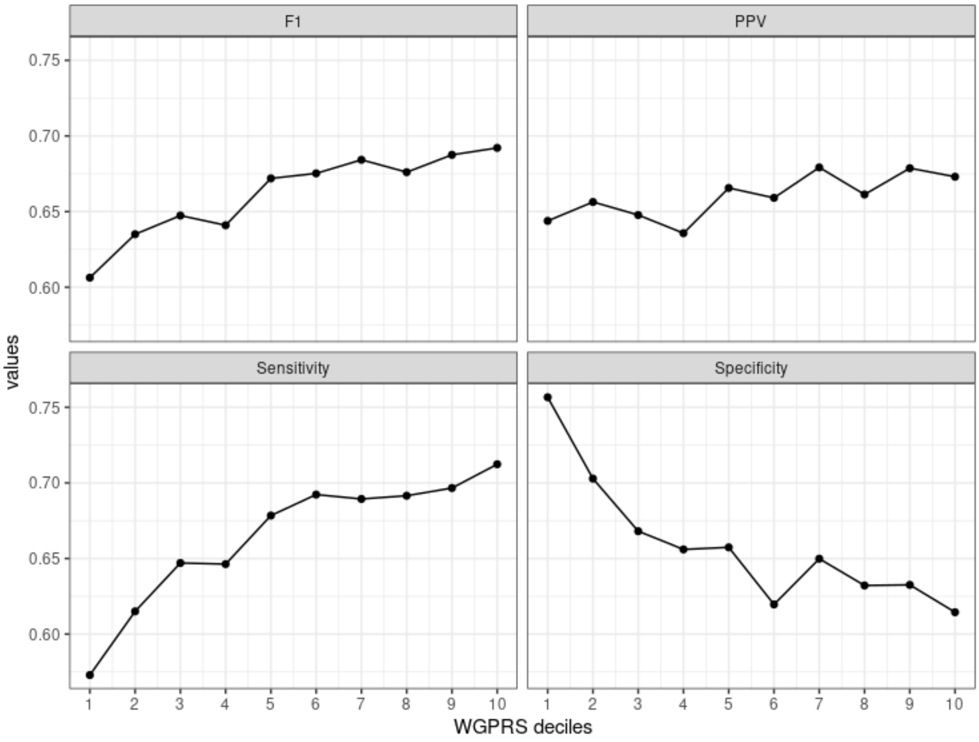

Supplementary Fig. 10: Predictive performance of the ClinWGPRS model, ranked by WGPRS decile. PPV = positive predictive value.

Supplementary Fig. 11

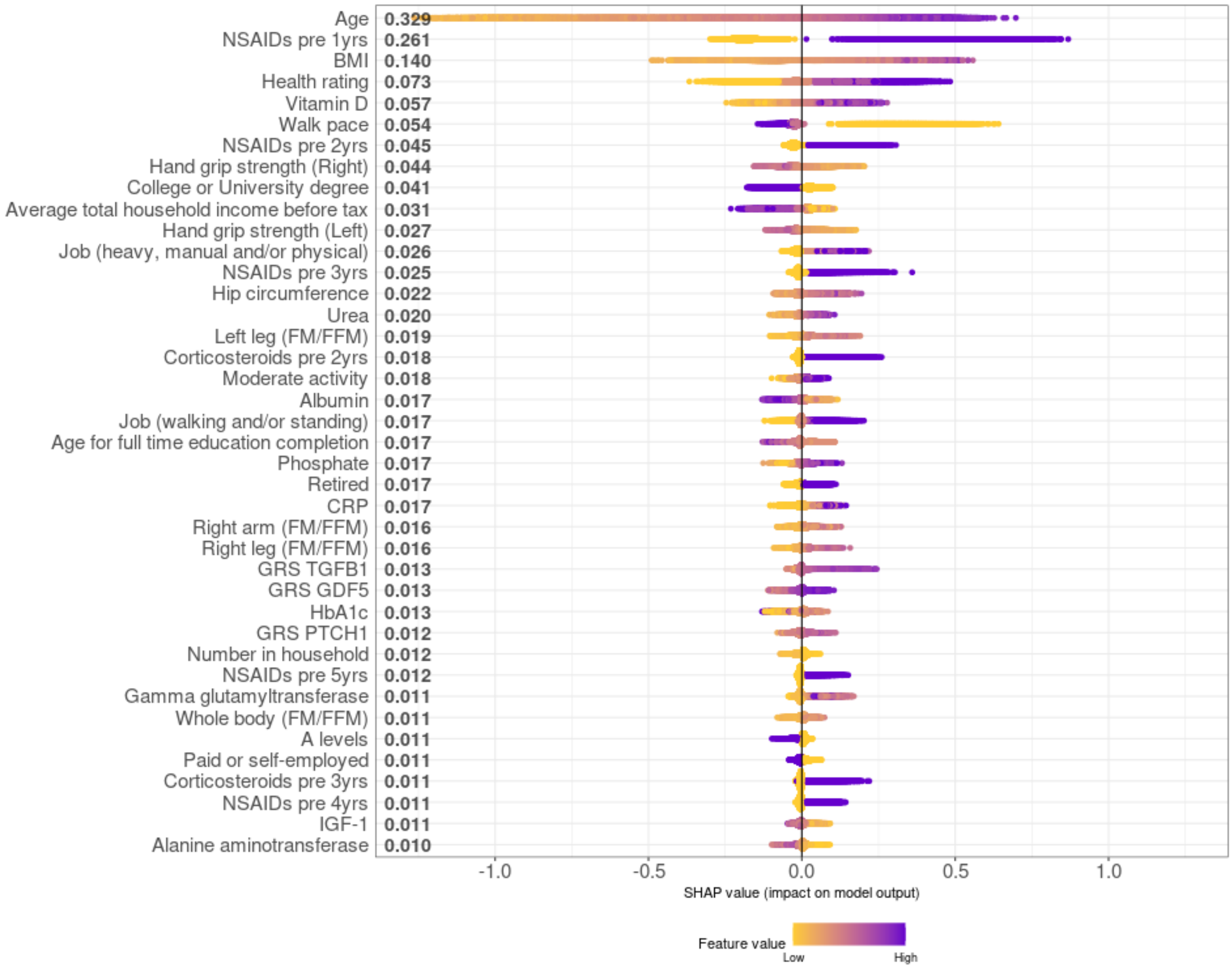

Supplementary Fig. 11: Ranked feature importance of ClinGRS (GRS obtained without proxy) model by SHAP additive explanations for top 40 predictive features in the model.

Supplementary Fig. 12

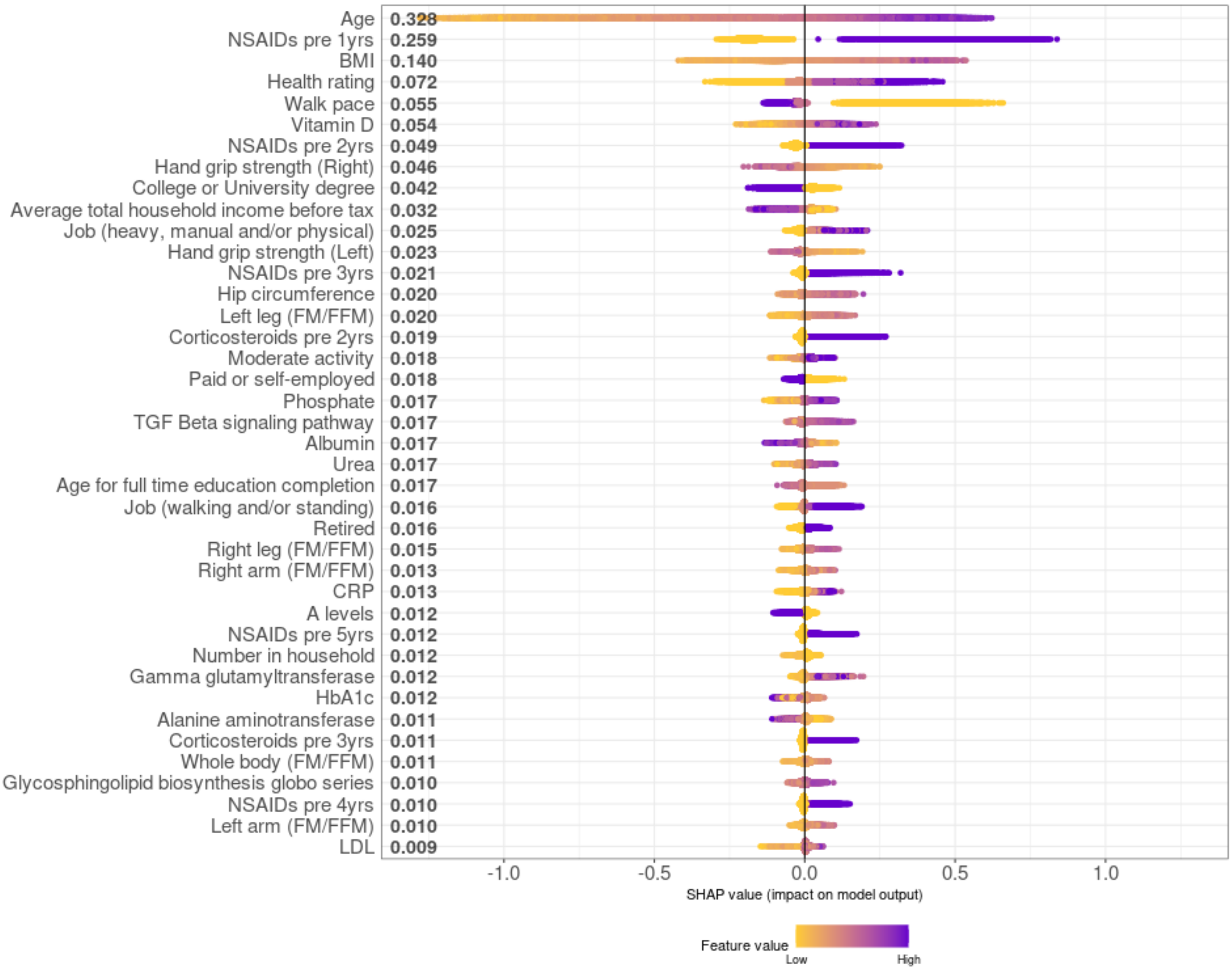

Supplementary Fig. 12: Ranked feature importance of ClinPath (PRS obtained without proxy) model by SHAP additive explanations for top 40 predictive features in the model.

Supplementary Fig. 13

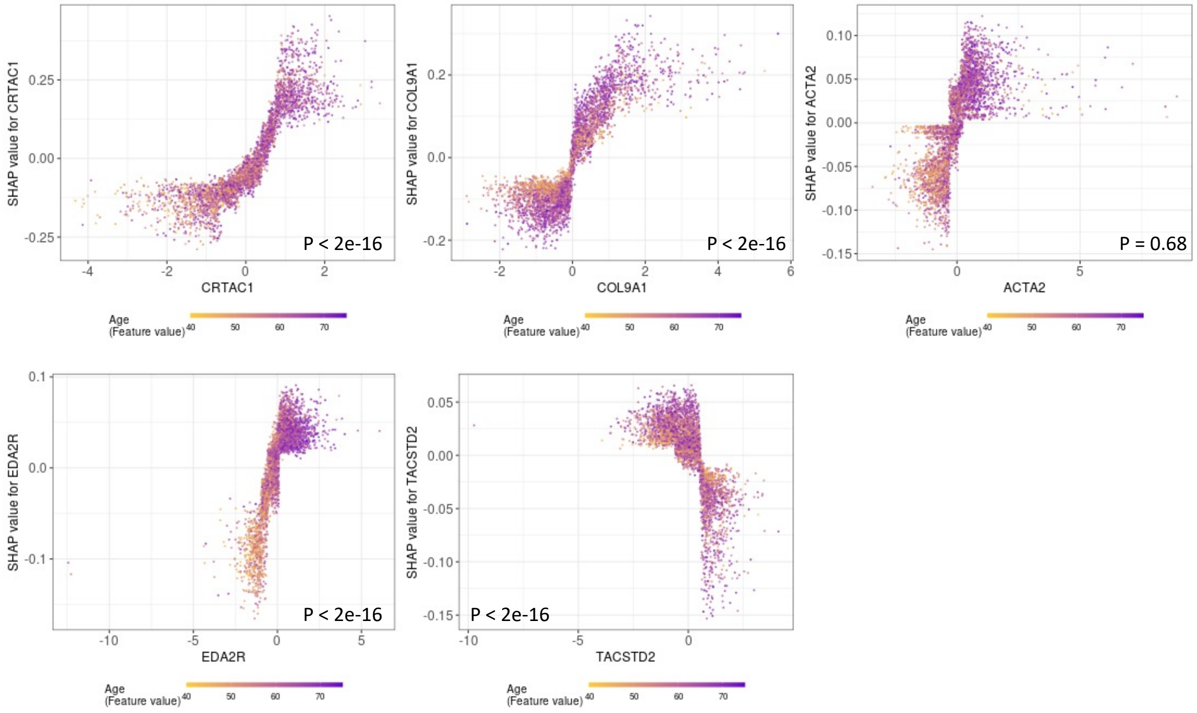

Supplementary Fig. 13: SHAP dependence plots between age and top five proteins (p-value from glm.anova).

NSAIDs prescriptions one year before OA diagnosis/proxy date have significant interaction dependencies with COL9A1 (p<2e-16), ACTA2(p=2.8e-4), EDA2R (p<2e-16), TACSTD2 (p=2.8e-5) (plots not shown). BMI had significant interaction dependencies with CRTAC1 (p=6e-4), ACTA2 (p=0.01), EDA2R (p<2e-16) and TACSTD2 (p=1.6e-9) (plots not shown).

Supplementary Fig. 14

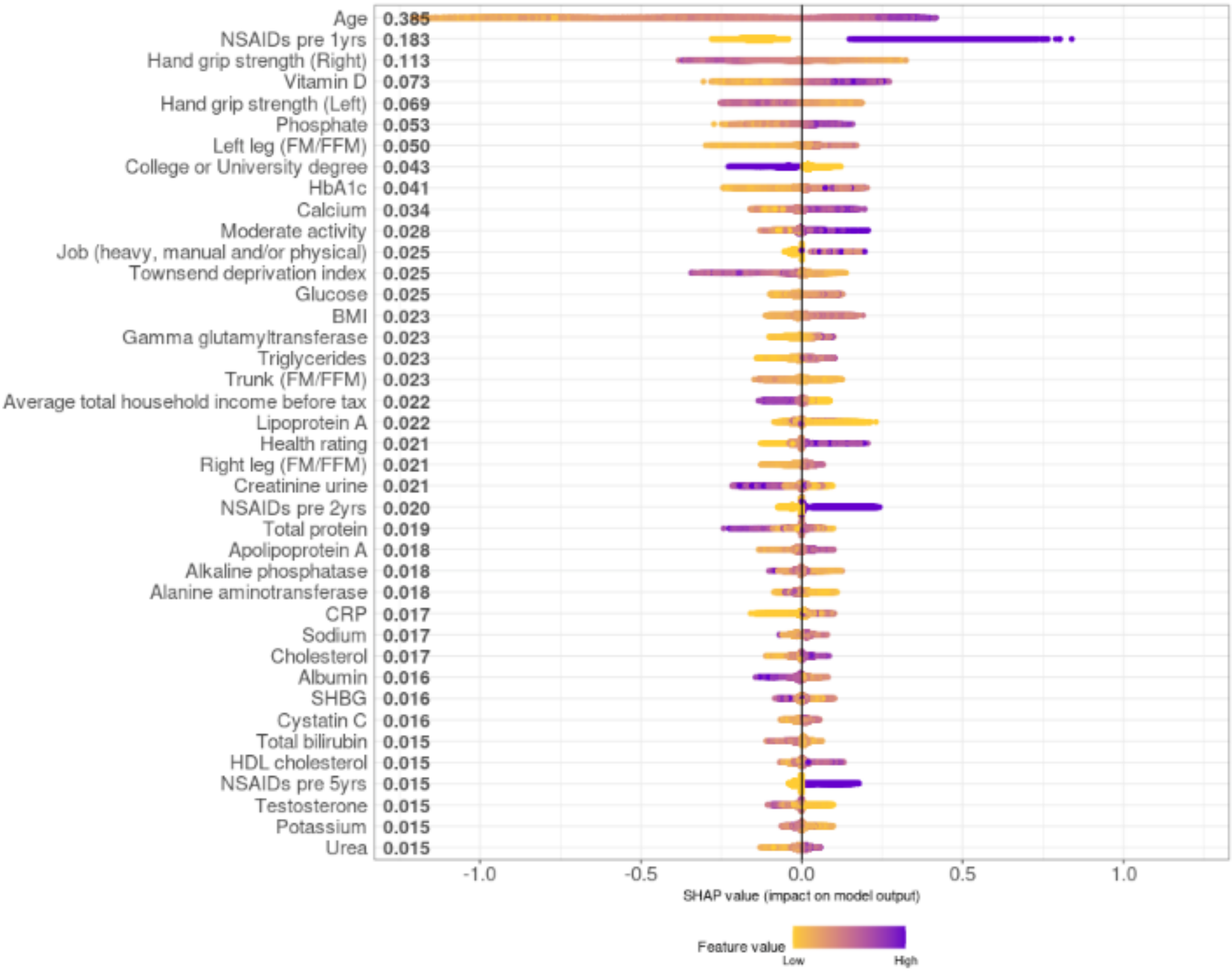

Supplementary Fig. 14: Ranked feature importance of Clin model only using OA cases diagnosed in the arm by SHAP additive explanations for top 40 predictive features in the model.

Supplementary Fig. 15

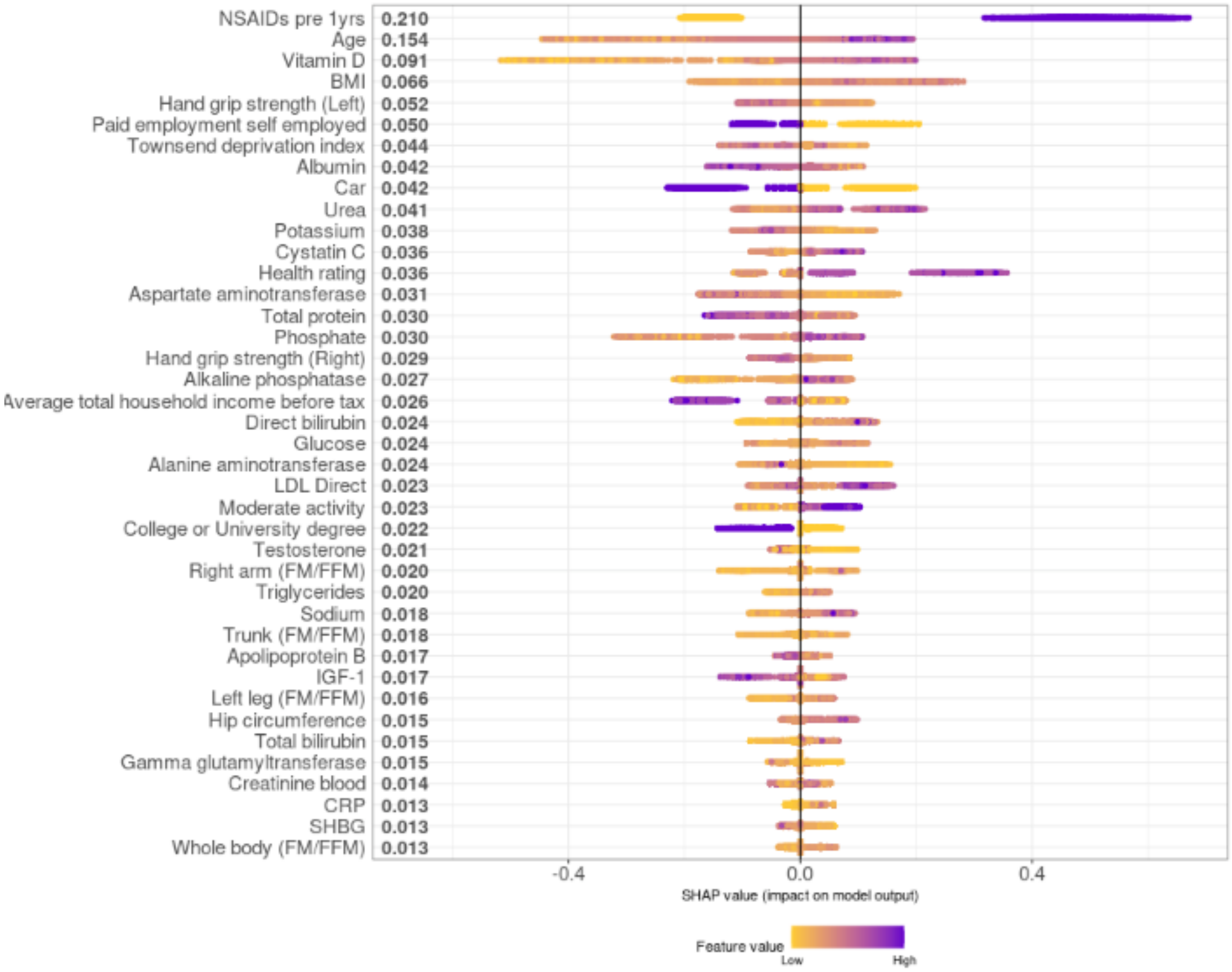

Supplementary Fig. 15: Ranked feature importance of Clin model only using OA cases diagnosed in the foot by SHAP additive explanations for top 40 predictive features in the model.

Supplementary Fig. 16

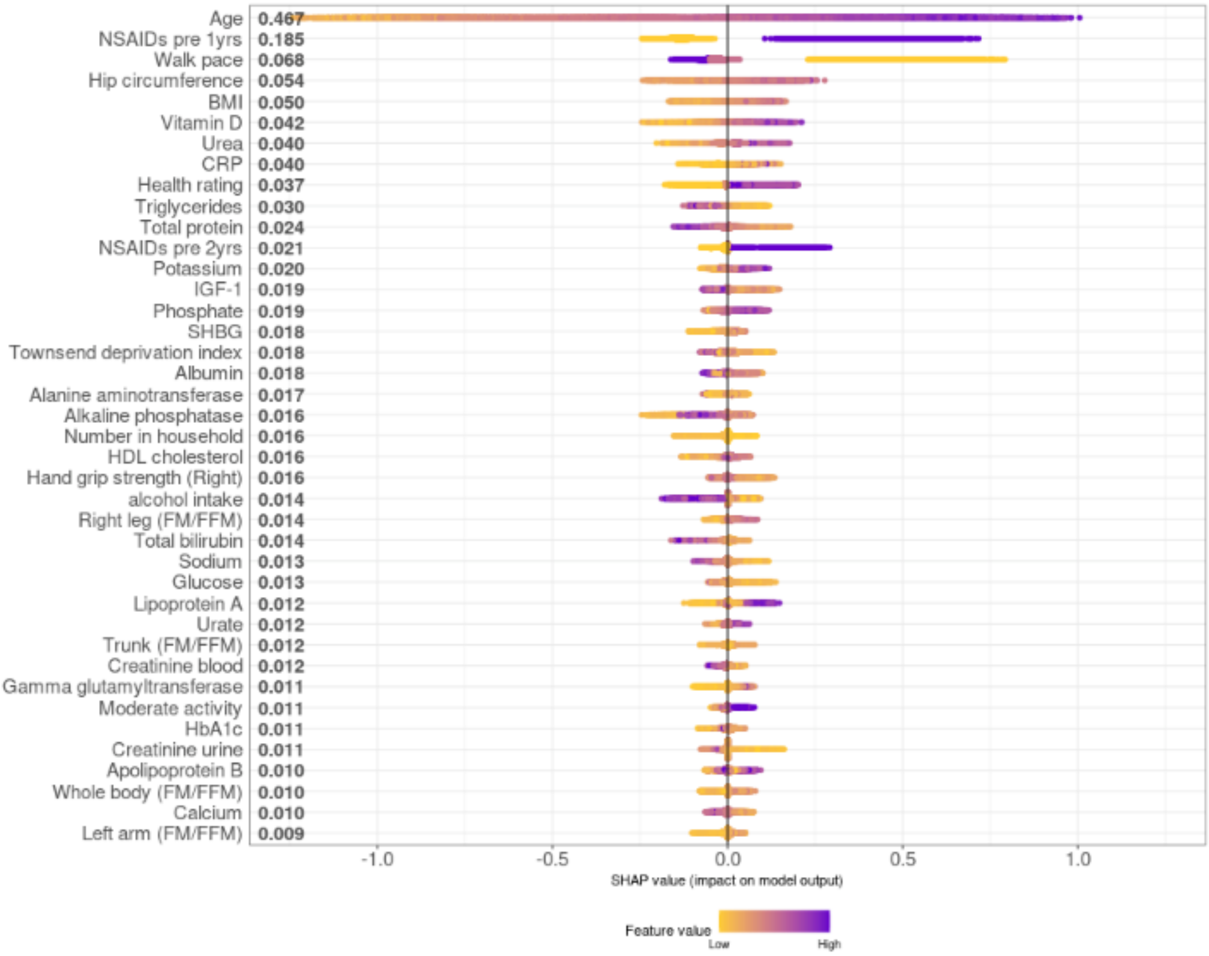

Supplementary Fig. 16: Ranked feature importance of Clin model only using OA cases diagnosed in the hip by SHAP additive explanations for top 40 predictive features in the model.

Supplementary Fig. 17

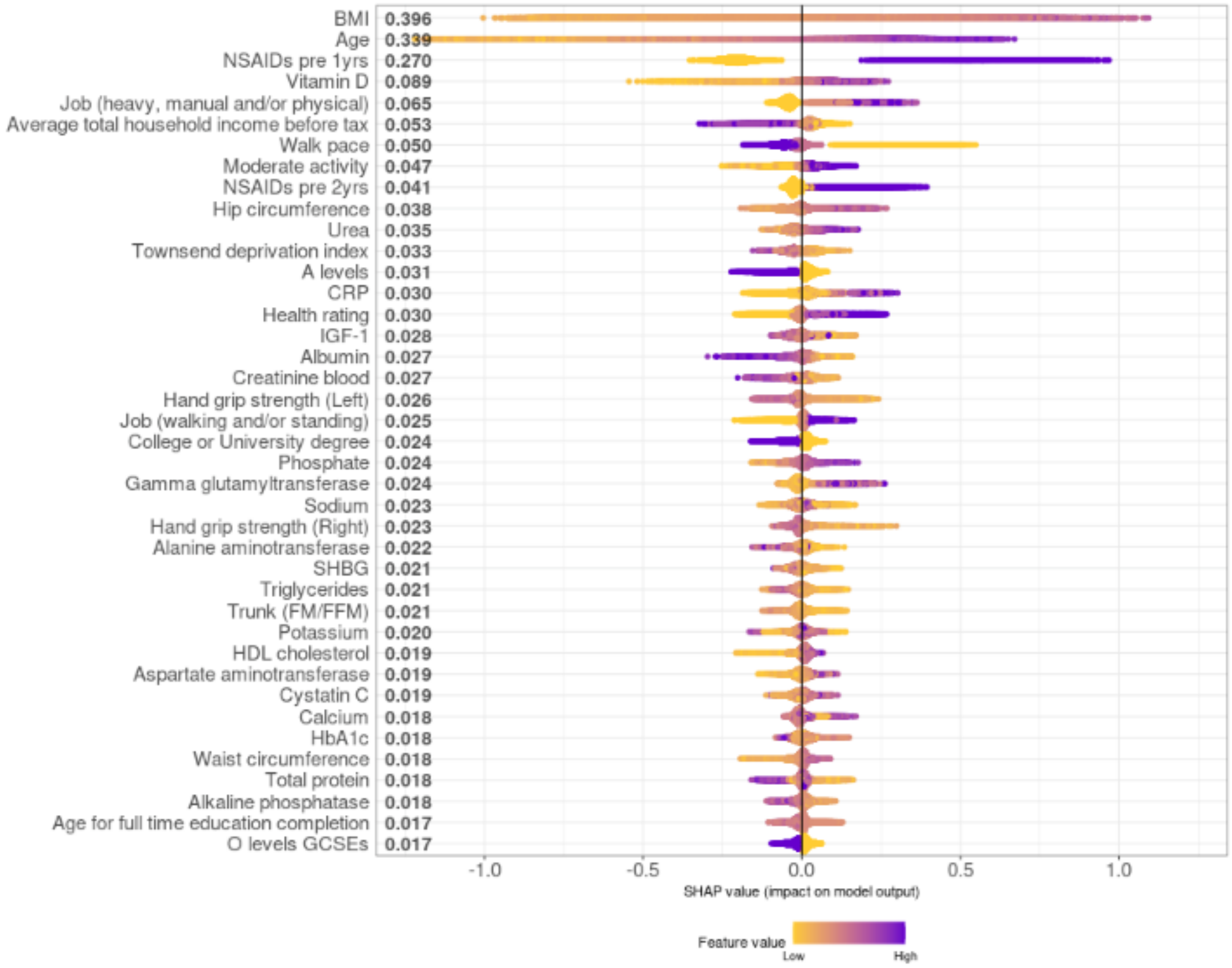

Supplementary Fig. 17: Ranked feature importance of Clin model only using OA cases diagnosed in the knee by SHAP additive explanations for top 40 predictive features in the model.

Supplementary Fig. 18

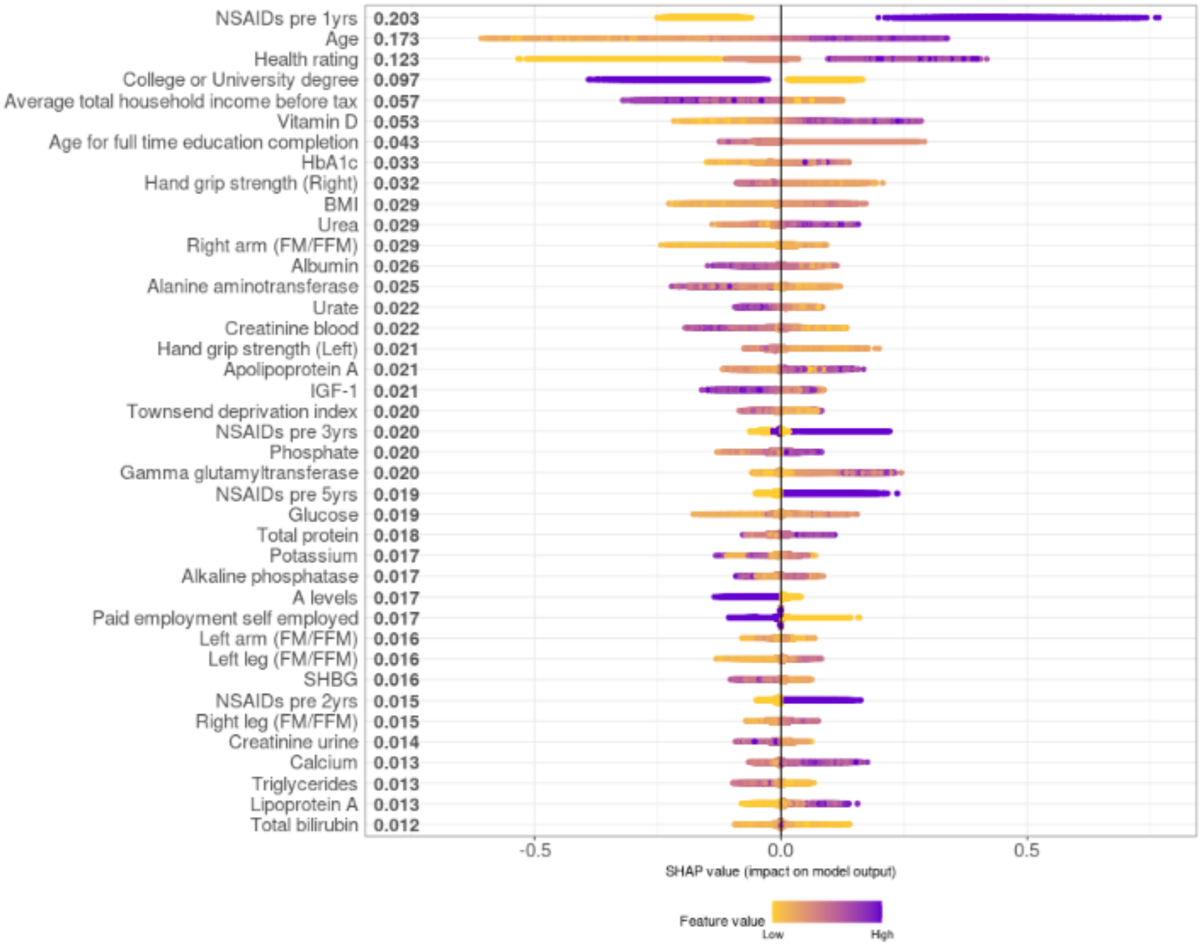

Supplementary Fig. 18: Ranked feature importance of Clin model only using OA cases diagnosed in the spine by SHAP additive explanations for top 40 predictive features in the model.

Supplementary Tables

Supplementary Table 1

Supplementary Table 1: Robustness and stability of Clin model across 100 random model initialisations (cross-validation splits and model parameters). To test stability of the model, the labels of OA cases and controls were randomly permuted and tested with the same 100 model initialisations. ROC-AUC is given as cross-validated mean ROC-AUC and 95%CI on the test set. PPV = positive predictive value. NPV = Negative predictive value.

	ROC-AUC	Sensitivity	PPV	Specificity	NPV
Robustness of Clin model across 100 model initialisations	0.72 (0.72 - 0.72)	0.67 (0.67 – 0.67)	0.65 (0.65 – 0.66)	0.65 (0.65 – 0.65)	0.66 (0.66 – 0.66)
Clin model across 100 model initialisations on permuted outcome label	0.50 (0.50 – 0.51)	0.49 (0.49 – 0.49)	0.50 (0.50 – 0.50)	0.51 (0.51 – 0.51)	0.50 (0.50 – 0.50)

Supplementary Table 2

Supplementary Table 2: Sensitivity analysis of predictive performance (ROC-AUC) of the Clin model retrained on subsets of individuals with available omics. ROC-AUC is given as cross-validated mean ROC-AUC and 95%CI on the test set.

ClinSNP	ClinGRS (proxy)	ClinGRS (noproxy)	ClinPath (proxy)	ClinPath (noproxy)	ClinMet	ClinPro
0.73 (0.72 – 0.73)	0.72 (0.71 – 0.72)	0.72 (0.71 – 0.72)	0.72 (0.71 – 0.72)	0.72 (0.71 – 0.72)	0.72 (0.71 – 0.73)	0.69 (0.65 – 0.73)

Supplementary Table 3

Supplementary Table 3: ClinWGPRS, ClinGRS and ClinPath models where genetic features are adjusted with 10 principal components of population substructure and batch corrected. Ranked feature importance by SHAP additive explanations for top 40 predictive features for these models are not shown as this analysis was conducted as a sensitivity check of the WGPRS and gene-GRS/pathway-PRS. ROC-AUC is given as cross-validated mean ROC-AUC and 95%CI on the test set.

	ClinWGPRS	ClinGRS (proxy)	ClinGRS (noproxy)	ClinPath (proxy)	ClinPath (noproxy)
ROC-AUC	0.72 (0.72 – 0.73)	0.72 (0.71 – 0.72)	0.72 (0.71 – 0.72)	0.72 (0.71 – 0.72)	0.72 (0.71 – 0.72)
Ranking of features	WGPRS (ranked 9th most important feature)	TGFB1, GDF5, CAMKV	GDF5, TGFB1 ANKRD13D	TGF beta signaling pathway, adipocyte signaling pathway, cytokine-cytokine receptor interaction	TGF beta signaling pathway, Leishmania infection
