# Supplementary material for: Data-driven identification of predictive risk biomarkers for subgroups of osteoarthritis using interpretable machine learning
Source: Nat Commun. 2024 Apr 1;15:2817. doi: 10.1038/s41467-024-46663-4 (PMC10985086; doi:10.1038/s41467-024-46663-4)
Supplement: Supplementary file 3 — Description of Additional Supplementary Files [file 41467_2024_46663_MOESM3_ESM.pdf]

Description of Additional Supplementary Files

Supplementary Data 1: Osteoarthritis clinical diagnosis codes in Read v2, Read CTV3, ICD-9 and ICD-10 format.

Supplementary Data 2: Missingness percentages of clinical data variables used in the Clin model.

Supplementary Data 3: Full list of differentially expressed proteins per cluster (for all samples, only cases, or only controls in each cluster).

Supplementary Data 4: Assessment centre input features with full descriptions.

Supplementary Data 5: Primary care biomarker Read (v2 / CTV3) codes by biomarker name and class.

Supplementary Data 6: Primary care medication codes (Read v2 / BNF / DM+D) by drug name, class, and associated disease of interest.

Supplementary Data 7: TRIPOD Checklist: Prediction Model Development.
