# Supplementary material for: Data-driven identification of predictive risk biomarkers for subgroups of osteoarthritis using interpretable machine learning
Source: Nat Commun. 2024 Apr 1;15:2817. doi: 10.1038/s41467-024-46663-4 (PMC10985086; doi:10.1038/s41467-024-46663-4)
Supplement: Supplementary file 10 — Supplementary Data 7 [file 41467_2024_46663_MOESM10_ESM.pdf]

TRIPOD Checklist: Prediction Model Development

Page numbers based on document with no markups

Section/Topic	Item	Checklist Item	Page
**Title and abstract**			
Title	1	Identify the study as developing and/or validating a multivariable prediction model, the target population, and the outcome to be predicted.	Section: Title
Abstract	2	Provide a summary of objectives, study design, setting, participants, sample size, predictors, outcome, statistical analysis, results, and conclusions.	Section: Abstract
**Introduction**			
Background and objectives	3a	Explain the medical context (including whether diagnostic or prognostic) and rationale for developing or validating the multivariable prediction model, including references to existing models.	Section: Introduction
	3b	Specify the objectives, including whether the study describes the development or validation of the model or both.	Section: Introduction
**Methods**			
Source of data	4a	Describe the study design or source of data (e.g., randomized trial, cohort, or registry data), separately for the development and validation data sets, if applicable.	Sections: Methods/UK Biobank + Methods/Pre-processing of electronic health record data + Methods/OA study and validation populations + Fig 1 + Sup Fig 1
	4b	Specify the key study dates, including start of accrual; end of accrual; and, if applicable, end of follow-up.	Sections: Results/OA study population + Methods/UK Biobank + Methods/OA study and validation populations + Fig 1 + Supp. Fig 1
Participants	5a	Specify key elements of the study setting (e.g., primary care, secondary care, general population) including number and location of centres.	Sections: Results/OA study population + Methods/UK Biobank + Methods/Pre-processing of assessment centre data + Fig 1, 2
	5b	Describe eligibility criteria for participants.	Sections: Results/OA study population + Methods/UK Biobank + Methods/Pre-processing of assessment centre data + Supp. Fig 1 + Methods/OA study and validation populations
	5c	Give details of treatments received, if relevant.	Section: Methods/Pre-processing of electronic health record data + Supp. Data 6
Outcome	6a	Clearly define the outcome that is predicted by the prediction model, including how and when assessed.	Sections: Methods/OA study and validation populations + Results/OA study population
	6b	Report any actions to blind assessment of the outcome to be predicted.	Section: Methods/OA study and validation populations + Results/OA study population
Predictors	7a	Clearly define all predictors used in developing or validating the multivariable prediction model, including how and when they were measured.	Methods sections: UK Biobank, Pre-processing of assessment centre data + Pre-processing of electronic health record data + Supp. Data 4
	7b	Report any actions to blind assessment of predictors for the outcome and other predictors.	Sections: Methods/OA study and validation populations + Results/OA study population
Sample size	8	Explain how the study size was arrived at.	Supp. Data 1 + Sections: Results/OA study population Methods/OA study and validation populations
Missing data	9	Describe how missing data were handled (e.g., complete-case analysis, single imputation, multiple imputation) with details of any imputation method.	Supp. Data 2 + Section: Methods/Machine learning models
Statistical analysis methods	10a	Describe how predictors were handled in the analyses.	Sections: Methods/Pre-processing of assessment centre data + Methods/Pre-processing of electronic health record data
	10b	Specify type of model, all model-building procedures (including any predictor selection), and method for internal validation.	Sections: Methods/Machine learning models + Methods/Model interpretation + Methods/Cluster analyses
	10d	Specify all measures used to assess model performance and, if relevant, to compare multiple models.	Sections: Methods/Machine learning models + Methods/Model interpretation + Methods/Cluster analyses
Risk groups	11	Provide details on how risk groups were created, if done.	Sections: Methods/Cluster analyses + Results/Precision sub-groups of OA
**Results**			
Participants	13a	Describe the flow of participants through the study, including the number of participants with and without the outcome and, if applicable, a summary of the follow-up time. A diagram may be helpful.	Sections: Results/OA study population + Supp. Fig 1
	13b	Describe the characteristics of the participants (basic demographics, clinical features, available predictors), including the number of participants with missing data for predictors and outcome.	Sections: Results/OA study population + Supp. Fig 1 + Supp. Data 2
Model development	14a	Specify the number of participants and outcome events in each analysis.	Sections: Results/OA study population + Fig 1, 7 + Supp. Fig 1
	14b	If done, report the unadjusted association between each candidate predictor and outcome.	NA
Model specification	15a	Present the full prediction model to allow predictions for individuals (i.e., all regression coefficients, and model intercept or baseline survival at a given time point).	Sections: Results/Risk modelling + Results/Prediction of OA from five-year multi-modal clinical data + Results/Population risk biomarkers predictive of OA risk + Results/Precision sub-groups of OA + Results/Personalised risk biomarkers of OA + Results/Multi-omics OA risk biomarkers + Results/OA risk biomarker heterogeneity across joints
	15b	Explain how to the use the prediction model.	Sections: Results/Risk modelling + Results/Prediction of OA from five-year multi-modal clinical data + Results/Population risk biomarkers predictive of OA risk + Results/Precision sub-groups of OA + Results/Personalised risk biomarkers of OA + Results/Multi-omics OA risk biomarkers + Results/OA risk biomarker heterogeneity across joints + Methods/Machine learning models + Methods/Model interpretation
Model performance	16	Report performance measures (with CIs) for the prediction model.	Sections: Results/Prediction of OA from five-year multi-modal clinical data + Results/OA risk biomarker heterogeneity across joints + Fig 3
**Discussion**			
Limitations	18	Discuss any limitations of the study (such as nonrepresentative sample, few events per predictor, missing data).	Section: Discussion
Interpretation	19b	Give an overall interpretation of the results, considering objectives, limitations, and results from similar studies, and other relevant evidence.	Section: Discussion
Implications	20	Discuss the potential clinical use of the model and implications for future research.	Section: Discussion
**Other information**			
Supplementary information	21	Provide information about the availability of supplementary resources, such as study protocol, Web calculator, and data sets.	Sections: Data Availability + Code Availability
Funding	22	Give the source of funding and the role of the funders for the present study.	Section: Acknowledgements

We recommend using the TRIPOD Checklist in conjunction with the TRIPOD Explanation and Elaboration document.
