# Supplementary material for: Data-driven identification of predictive risk biomarkers for subgroups of osteoarthritis using interpretable machine learning
Source: Nat Commun. 2024 Apr 1;15:2817. doi: 10.1038/s41467-024-46663-4 (PMC10985086; doi:10.1038/s41467-024-46663-4)
Supplement: Supplementary file 11 — Reporting Summary [file 41467_2024_46663_MOESM11_ESM.pdf]

nature portfolio

Corresponding author(s): Ramneek Gupta

Last updated by author(s): Feb 12, 2024

Reporting Summary

Nature Portfolio wishes to improve the reproducibility of the work that we publish. This form provides structure for consistency and transparency in reporting. For further information on Nature Portfolio policies, see our Editorial Policies and the Editorial Policy Checklist.

Statistics

For all statistical analyses, confirm that the following items are present in the figure legend, table legend, main text, or Methods section.

n/a	Confirmed	
☐	☒	The exact sample size (*n*) for each experimental group/condition, given as a discrete number and unit of measurement
☐	☒	A statement on whether measurements were taken from distinct samples or whether the same sample was measured repeatedly
☐	☒	The statistical test(s) used AND whether they are one- or two-sided<br>*Only common tests should be described solely by name; describe more complex techniques in the Methods section.*
☐	☒	A description of all covariates tested
☐	☒	A description of any assumptions or corrections, such as tests of normality and adjustment for multiple comparisons
☐	☒	A full description of the statistical parameters including central tendency (e.g. means) or other basic estimates (e.g. regression coefficient) AND variation (e.g. standard deviation) or associated estimates of uncertainty (e.g. confidence intervals)
☐	☒	For null hypothesis testing, the test statistic (e.g. *F*, *t*, *r*) with confidence intervals, effect sizes, degrees of freedom and *P* value noted<br>*Give P values as exact values whenever suitable.*
☒	☐	For Bayesian analysis, information on the choice of priors and Markov chain Monte Carlo settings
☐	☒	For hierarchical and complex designs, identification of the appropriate level for tests and full reporting of outcomes
☐	☒	Estimates of effect sizes (e.g. Cohen's *d*, Pearson's *r*), indicating how they were calculated

Our web collection on statistics for biologists contains articles on many of the points above.

Software and code

Policy information about availability of computer code

Data collection	To further extract and process relevant field and clinical codes of data from the UK Biobank R v4.2.0 was used.<br>For genetic data data processing, PLINK (v2.00a3LM) and PRSice (v.2.3.3) were used.
Data analysis	All analyses were performed on publicly available software, and all parameters are provided in methods wherever relevant. The code used for this study was tailored to the data and the fields of the UK Biobank data and is no use as a standalone without access to the data (requires UK Biobank access). However, code to reproduce the study, as well as files describing input data formats, are provided in a git repository: https://github.com/novonordisk-research/xOAML. The authors welcome being contacted to provide more information to reproduce the results presented in this paper if needed.<br><br>Used public software. All are cited in methods:<br>PLINK (v.2.00a3LM)<br>PRSice (v.2.3.3)<br>R (v.4.2.0)<br>xgboost (v.1.6.0.1)<br>pROC (v.1.18)<br>caret (v.6.0-93)<br>yardstick (v.1.1.0)<br>SHAPforxgboost (v.0.1.1)<br>shapviz (v.0.4.1)<br>Seurat85 (v.4.3.0)<br>chooseR (v.12062020)

ComplexHeatmap (v.2.13.1)
SkopeRules (v.1.0.1)

For manuscripts utilizing custom algorithms or software that are central to the research but not yet described in published literature, software must be made available to editors and reviewers. We strongly encourage code deposition in a community repository (e.g. GitHub). See the Nature Portfolio guidelines for submitting code & software for further information.

Data

Policy information about availability of data

All manuscripts must include a data availability statement. This statement should provide the following information, where applicable:

- Accession codes, unique identifiers, or web links for publicly available datasets
- A description of any restrictions on data availability
- For clinical datasets or third party data, please ensure that the statement adheres to our policy

UK Biobank is available to researchers following application to the UK Biobank database (https://www.ukbiobank.ac.uk/enable-your-research/apply-for-access). All field IDs and clinical codes used for extraction of data have been provided in Supplementary Data. The use of UK Biobank for this study was performed under research application numbers 53639 and 65851.

The OA GWAS summary statistics files used to generate the genetic risk scores were created as part of a published study by Boer et al, Cell 2021 (https://pubmed.ncbi.nlm.nih.gov/34450027/).

KEGG pathways obtained via the Molecular Signature Database (MsigDB; v2022.1, https://www.gsea-msigdb.org/gsea/msigdb).

The gene regions were defined using GENCODE annotations (v.43lift37); only protein-coding genes were considered for analyses (https://www.gencodegenes.org/human/release_43lift37.html).

Source data that is not patient-sensitive data are provided with this paper to reproduce figures and tables in the main manuscript in https://github.com/novonordisk-research/xOAML, including links to any publicly-available datasets used in the analyses. In the case of individual-level sensitive data from UK Biobank mock data describing input file formats is provided alongside code used to reproduce the figures and tables.

Research involving human participants, their data, or biological material

Policy information about studies with human participants or human data. See also policy information about sex, gender (identity/presentation), and sexual orientation and race, ethnicity and racism.

Reporting on sex and gender	The study only reports on biological Sex (biological attribute). Sex indicates Male or Female. Sex of participants in UK biobank were acquired from central registry at recruitment, but in some cases updated by the participant. Hence this field may contain a mixture of the sex the NHS had recorded for the participant and self-reported sex. For the study, we used the following field; https://biobank.ctsu.ox.ac.uk/crystal/field.cgi?id=31
Reporting on race, ethnicity, or other socially relevant groupings	Several potential risk biomarkers were explored using an unbiased data-driven machine learning approach to rank to most other socially relevant important features for prediction risk of developing osteoarthritis.<br>We accounted for genetic ethnicity based on principal components of genetic data ancestry, but did not interpret these as socially constructed variables. For the clinical model, we included high-level information on whether individuals had a European genetic ancestry (yes/no) and for subsets of models including genetic-based features we also included 10 principal components of genetically determined population substructure.<br>We used socio-economic variables that are relevant to disease prediction, such as Townsend deprivation index (as given by UK Biobank) features of self-reported touch screen questionaire data on housing situation, educational background, transportation means to work, physical disabilities and type of job (such as including heavy lifting or working night shift); however they were not used as proxy for other socially constructed variables.<br>We controlled for confounding variables by ensuring short-length of decision trees (making sure only the most relevant features for prediction were selected). Furthermore, the XGBoost model included subsampling of 80% of random uniformly selected samples as well as features available during model training.<br>All clinical codes (read2, ctv3, ICD-9, ICD-10, bnf codes and UK biobank field codes) used for extraction of data in this study is provided in Supplementary Files including details of which biomarkers was extracted.
Population characteristics	We described the population characteristics between people developing osteoarthritis and those that did not develop osteoarthritis by the the most commonly epidemiological osteoarthritis risk factors including age, sex and BMI across the study population and the validation population (See Fig 1B)
Recruitment	For this study, we have not been involved in recruitment of study participants, but used UK Biobank open research resource under application numbers 53639 and 65851.<br>Information obtained from UK Biobank on recruitment:<br>UK Biobank holds an unprecedented amount of data on half a million participants aged 40-69 years (with a roughly even number of men and women) recruited between 2006 and 2010 throughout the UK.All participants in UK Biobank were recruited through assessment centres, designed specifically for this purpose (a map of the 22 assessment centres is provided in the Essential Information section of the Showcase). Data collected at the assessment visit included information ona participant's health and lifestyle, hearing and cognitive function, collected through a touchscreen questionnaire and brief verbal interview. A range of physical measurements were also performed, which included: blood pressure; arterial stiffness; eye measures (visual acuity, refractometry, intraocular pressure, optical coherence tomography); body composition measures (including impedance); hand-grip strength; ultrasound bone densitometry; spirometry; and an exercise/fitness test with ECG. Samples of blood, urine and saliva were also collected.

	Text from UK Biobank recruitment information obtained from: https://biobank.ctsu.ox.ac.uk/~bbdatan/ShowcaseUserGuide.pdf
Ethics oversight	The UK biobank has ethical approval from the North West Multi-centre Research Ethics Committee (REC reference number: 16/NW/0274). Written informed consent was obtained for all UK biobank study participants.

Note that full information on the approval of the study protocol must also be provided in the manuscript.

Field-specific reporting

Please select the one below that is the best fit for your research. If you are not sure, read the appropriate sections before making your selection.

☒ Life sciences ☐ Behavioural & social sciences ☐ Ecological, evolutionary & environmental sciences

For a reference copy of the document with all sections, see nature.com/documents/nr-reporting-summary-flat.pdf

Life sciences study design

All studies must disclose on these points even when the disclosure is negative.

Sample size	The UK Biobank is a population-based cohort study with health information from assessments at the time of recruitment (2006–2010) and linkage to electronic health records (EHR) of individuals in the UK (N=502,476). We identified 103,086 patients with an OA diagnosis from EHR data (~21% of all UK Biobank participants, Supplementary Fig. 1). In total, 55,628 OA diagnoses were identified from primary care settings (general practices, follow-up until 09/2017) and 49,318 OA diagnoses from secondary care settings (hospital inpatient data, follow-up until 03/2017). Clinical codes of OA diagnoses are given in Supplementary File 1 (primary care: Read v2 and CTV3/Read v3, secondary care: ICD-9 or ICD-10).<br><br>Primary healthcare data is available for ~45% of the UK Biobank cohort which enabled capture of longitudinal data for a subset of patients that were diagnosed with OA (N=67,772). An equal number of control participants who were never diagnosed with OA in the available EHR study period were identified (N=67,772). Controls were randomly selected and date-matched with the OA diagnosis dates for case patients. Cases and controls were then filtered for those with an OA diagnosis/matched index date a maximum of five years after the UK Biobank recruitment assessment centre. We focussed our study on the diagnosis of OA up to five years after the assessment centre. This was to capture the risk biomarkers that are predictive of OA diagnosis in the focussed period of five years prior to diagnosis, when patients are at high-risk, and a potential window to explore for preventative interventions with the deep phenotyping of the aging population. Controls were required to have observational data and no death registered during the five years prior to the index date (study period: 06/2006–09/2015). This resulted in a total of 19,120 patients with diagnosed OA and 19,252 controls included in the analysis (Fig. 1A, Supplementary Fig. 1). Additionally an independent hold-out validation cohort included 7,341 patients diagnosed with OA and 5,999 controls.<br><br>Small error bars were observed for performance estimates indicating sufficient sample size for stable model performance. An analysis of the confidence interval for AUROC, given the sample size for our study (N = 38,372), indicated that the lower bound for the CI for AUROC is 0.7149 and the upper bound is 0.7251 (https://sample-size.net/confidence-interval-for-auroc-given-n/).
Data exclusions	This is an observational real-world data study on osteoarthritis.<br>Individuals were excluded from analysis if;<br>- Study participants had been diagnosed with osteoarthrtitis but had a missing date of diagnosis in their electronic health records<br>- Study participants younger than 18 years at the time of osteoarthritis diagnosis<br>- no death registration within the study period<br>- Primary care data not available<br>All data exclusions are described in Online Methods as well as outlined in Sup Fig 1.
Replication	The findings from the machine learning models were summarised into explainable subgroups with differing risk of developing osteoarthrtitis. To validate the potential clinical value of these rules associated to the identfied subgroups of osteoarthritis, we applied them to an independent hold-out population (with similar case/control definitions, and with cases being diagnosed more than five years after the assessment center visit; 7,341 cases and 5,999 controls). For this validation cohort, the time between data collection at the assessment centre and OA diagnosis was more than five years.<br>No replication has been sought in external cohorts due to the study design being specific to UK Biobank.
Randomization	This is an observational real-world data study and no randomisation has been performed.
Blinding	Not relevant due to the retrospective study design using supervised machine learning approaches.<br><br>In a retrospective study, the intervention or exposure would likely have been administered outside the context of a research study; therefore, blinding of the intervention/exposure may not be applicable. - Viswanathan M, Berkman ND, Dryden DM, et al. Assessing Risk of Bias and Confounding in Observational Studies of Interventions or Exposures: Further Development of the RTI Item Bank [Internet]. Rockville (MD): Agency for Healthcare Research and Quality (US); 2013 Aug. Appendix A, Approaches to Assessing the Risk of Bias in Studies. Available from: https://www.ncbi.nlm.nih.gov/books/NBK154465/

Reporting for specific materials, systems and methods

We require information from authors about some types of materials, experimental systems and methods used in many studies. Here, indicate whether each material, system or method listed is relevant to your study. If you are not sure if a list item applies to your research, read the appropriate section before selecting a response.

Materials & experimental systems

n/a	Involved in the study	
☒	☐	Antibodies
☒	☐	Eukaryotic cell lines
☒	☐	Palaeontology and archaeology
☒	☐	Animals and other organisms
☒	☐	Clinical data
☒	☐	Dual use research of concern
☒	☐	Plants

Methods

n/a	Involved in the study	
☒	☐	ChIP-seq
☒	☐	Flow cytometry
☒	☐	MRI-based neuroimaging

Plants

Seed stocks

Report on the source of all seed stocks or other plant material used. If applicable, state the seed stock centre and catalogue number. If plant specimens were collected from the field, describe the collection location, date and sampling procedures.

Novel plant genotypes

Describe the methods by which all novel plant genotypes were produced. This includes those generated by transgenic approaches, gene editing, chemical/radiation-based mutagenesis and hybridization. For transgenic lines, describe the transformation method, the number of independent lines analyzed and the generation upon which experiments were performed. For gene-edited lines, describe the editor used, the endogenous sequence targeted for editing, the targeting guide RNA sequence (if applicable) and how the editor was applied.

Authentication

Describe any authentication procedures for each seed stock used or novel genotype generated. Describe any experiments used to assess the effect of a mutation and, where applicable, how potential secondary effects (e.g. second site T-DNA insertions, mosiacism, off-target gene editing) were examined.
